# Differential MicroRNA Expression Involved in Endometrial Receptivity of Goats

**DOI:** 10.3390/biom11030472

**Published:** 2021-03-22

**Authors:** Xupeng Zang, Chen Zhou, Wenjing Wang, Jianyu Gan, Yaokun Li, Dewu Liu, Guangbin Liu, Linjun Hong

**Affiliations:** 1 College of Animal Science, South China Agricultural University, Guangzhou 510642, China; xupeng_zang@stu.scau.edu.cn (X.Z.); czhou@stu.scau.edu.cn (C.Z.); wenjing_wang@stu.scau.edu.cn (W.W.); jygan@stu.scau.edu.cn (J.G.); ykli@scau.edu.cn (Y.L.); dwliu@scau.edu.cn (D.L.); 2 Lingnan Guangdong Laboratory of Modern Agriculture, Guangzhou 510642, China

**Keywords:** chi-miR-483, DTX3L, endometrium receptivity, goat, high-throughput sequencing, implantation

## Abstract

Endometrial receptivity represents one of the leading factors affecting the successful implantation of embryos during early pregnancy. However, the mechanism of microRNAs (miRNAs) to establish goat endometrial receptivity remains unclear. This study was intended to identify potential miRNAs and regulatory mechanisms associated with establishing endometrial receptivity through integrating bioinformatics analysis and experimental verification. MiRNA expression profiles were obtained by high-throughput sequencing, resulting in the detection of 33 differentially expressed miRNAs (DEMs), followed by their validation through quantitative RT-PCR. Furthermore, 10 potential transcription factors (TFs) and 1316 target genes of these DEMs were obtained, and the TF–miRNA and miRNA–mRNA interaction networks were constructed. Gene Ontology (GO) and Kyoto Encyclopedia of Genes and Genomes (KEGG) analyses indicated that these miRNAs were significantly linked to establishing endometrial receptivity. Moreover, the fluorescence in situ hybridization (FISH) analysis, dual-luciferase report assay, and immunohistochemistry (IHC) analysis corroborated that chi-miR-483 could directly bind to deltex E3 ubiquitin ligase 3L (DTX3L) to reduce its expression level. In conclusion, our findings contribute to a better understanding of molecular mechanisms regulating the endometrial receptivity of goats, and they provide a reference for improving embryo implantation efficiency.

## 1. Introduction

Embryo implantation is essential for the normal development of embryos in all mammals. Specifically in goat, it starts around day 15–16 of pregnancy [1]. Embryo implantation failure could result in high embryo mortality [2], affecting the litter size and adversely impacting livestock reproduction’s economic benefits. Endometrial receptivity represents one of the crucial elements influencing successful embryo implantation [3]. Receptive endometrium formation is a spatiotemporal process governed by numerous growth hormones, in addition to transcription factors and cytokines, such as estrogen and progesterone [4,5,6]. During this period, endometrium architecture and function have experienced remarkable variations, including the proliferation of endometrial stromal cells and differentiation of endometrial epithelial cells, making the uterus receptive to attachment and implantation by the embryo, thereby supporting the subsequent rapid embryo development [5,7]. Furthermore, the leading reason for frequent implantation failure in human-assisted reproductive technology is receptive endometrium dysfunction [8]. Consequently, supplementary research is required to acquire a more obvious precise molecular mechanism that regulates endometrial receptivity.

MicroRNAs (miRNAs) are small endogenous noncoding RNAs, processed from intergenic regions or introns of protein-coding RNAs, with a length of approximately 20–24 nucleotides [9,10]. Studies have revealed that miRNAs can target post-transcriptional messenger RNA (mRNA) through epigenetic modification by binding to complementary sites within 3′-untranslated regions (3′UTRs), thereby degrading or inhibiting its translation and regulating its expression [11,12]. In mammals, miRNAs play significant roles in numerous biological processes, such as intracellular signaling, cell proliferation, apoptosis, metabolism, organogenesis, and embryonic development [13,14,15,16]. MiRNAs have recently been reported to hold a critical function in regulating endometrium receptivity. For instance, miR-200c could hamper uterine receptivity formation by targeting fucosyltransferase 4 (FUT4) and α1.3-fucosylation on cell surface adhesion receptor CD44 [17], and miR-26a could regulate the expression of osteopontin (OPN), vascular endothelial growth factor (VEGF), cyclooxygenase-2 (COX-2), and prolactin (PRL) in endometrial cells to regulate the endometrial receptivity of dairy goats [18]. In addition, some miRNAs linked to endometrial receptivity have been identified in cows [19]. Nevertheless, no real clear information exists on the differences in miRNA expression in the endometrium between pregnant and nonpregnant goats, in addition to their role in endometrial receptivity.

Herein, we selected goat endometrium samples on day 16 of pregnancy (P16) of the same parity and nonpregnant goats on day 16 of the estrous cycle (C16). We conducted RNA sequencing of small RNAs existing in the goats’ endometrium on day 16 of pregnancy and nonpregnancy to identify miRNAs in the endometrium linked to endometrial receptivity. In line with our findings, an enhanced understanding of the molecular mechanisms regulating the endometrial receptivity of goats can be realized, thereby affording references for improving embryo implantation efficiency.

## 2. Materials and Methods

### 2.1. Animal and Tissue Collection

All the animals involved in this study were conducted in accordance with animal ethics guidelines and approved by the Animal Care and Use Committee of South China Agricultural University (permit number: SYXK-2014-0136). Six healthy and disease-free primiparous Chuanzhong black goats (*Capra hircus*) were acquired from Guangdong Wen’s Foodstuffs Group Co., Ltd. (Yunfu, China). The animals were randomly divided into the cyclic group (*n* = 3) and the pregnancy group (*n* = 3). After estrus, two artificial inseminations were performed on goats belonging to the pregnancy group. Goats were slaughtered at the local slaughterhouse on day 16 of the estrous cycle (C16) (*n* = 3) or pregnancy (P16) (*n* = 3). Pregnancy was confirmed by the presence of apparently normal filamentous conceptuses in uterine flushing [20]. For each animal, the uterus was quickly removed and transported to the laboratory in an icebox, and then it was opened longitudinally along the antimesometrial side. Approximately 1 cm^2^ endometrial tissue and uterine section (including myometrium and endometrium) samples were taken from the middle of each uterine horn at the antimesometrial side of the uterus. The uterine section samples were immediately fixed in 10% neutral-buffered formalin for 24 h followed by paraffin embedding (FFPE) for histology observation, RNA fluorescence in situ hybridization (FISH), and immunohistochemistry (IHC), and the endometrial samples were snap-frozen in liquid nitrogen and stored at −80 °C for RNA extraction.

### 2.2. Small RNA (sRNA) Library Construction and Sequencing

Total RNA was extracted from six goat endometrium samples using Trizol reagent (Invitrogen, Carlsbad, CA, USA) following the manufacturer’s instructions. RNA degradation and contamination were monitored on 1% agarose gels. RNA purity was checked using a NanoPhotometer^®^ spectrophotometer (IMPLEN, Westlake Village, CA, USA) at 260 and 280 nm, and the RNA integrity number (RIN) was assessed using the RNA Nano 6000 Assay Kit of the Agilent Bioanalyzer 2100 system (Agilent Technologies, Santa Clara, CA, USA). Approximately 3 μg of total RNA, with a RIN value above 8, was used as input material for the small RNA library according to the protocol of NEBNext^®^ Multiplex Small RNA Library Prep Set for Illumina^®^ (NEB, Ipswich, MA, USA). Three individual libraries under C16 condition were constructed by using three samples, followed byperforming the single-end sequencing (50 bp) on an Illumina Hiseq 2500 platform (Illumina, San Diego, CA, USA) at the Novogene (Beijing, China) according to the manufacturer’s recommended protocol; the P16 library was constructed in the same way as the C16 library.

### 2.3. Analysis of Sequencing Data

After high-throughput small RNA sequencing was completed, a filtering step was performed to remove reads containing poly-N (the proportion of N is greater than 10%), with 5′ adapter contaminants, without 3′ adapter or the insert tag, containing poly A, T, G, or C and low-quality reads (where the number of bases with a Phred value less than or equal to 20 accounts for more than 30% of the total number of read bases) from the raw data. Subsequently, the clean reads were mapped to the goat reference sequence by Bowtie (v.0.12.9) [21] without mismatch to analyze their expression and distribution on the reference. Then, the clean reads mapped to protein-coding genes, repeat sequences, ribosomal RNA (rRNA), transfer RNA (tRNA), small nuclear RNA (snRNA), and small nucleolar RNA (snoRNA), and small RNA tags were mapped to RepeatMasker (v.4.0.3) or Rfam database (ftp://selab.janelia.org/pub/Rfam/, accessed on 18 January 2021) [22]; alternatively, these types of data were removed. The available software tools mirDeep2 (v.2.0.0.5) [23] and srna-tools-cli (http://srna-tools.cmp.uea.ac.uk/, accessed on 18 January 2021) were used to acquire the potential miRNAs, and software tools miREvo (v.1.1) [24] and mirDeep2 were integrated to predict the novel miRNAs.

### 2.4. Identification of Differentially Expressed miRNAs (DEMs)

To compare differentially expressed miRNAs in the C16 and P16 endometrium of goats, the miRNA expression levels were normalized to calculate the expression of transcripts per million (TPM). Differential expression analysis of the two sets of libraries was performed using the DESeq R package (v.1.8.3) [25]. The *p*-values were adjusted using the Benjamini–Hochberg [26] method, and a corrected *p*-value of 0.05 was set as the threshold for significantly differential expression.

### 2.5. Potential Transcription Factors Prediction of DEMs

In order to explore potential transcription factors (TFs) of DEMs, we obtained the transcription factor binding site (TFBS) data from the JASPAR database (http://jaspar.genereg.net/, accessed on 18 January 2021) [27], and we expected the TFBSs to be preserved among vertebrates. The sequence information of DEMs was downloaded from the National Center for Biotechnology Information (NCBI) database, and sequences within 1 kb upstream of the transcription start site (TSS) were selected as the miRNA promoter region to predict the TF binding sites [28,29]. We employed TFBSTools software (v.1.20.0) to find binging sites [30]. To reduce the rate of false-positive prediction, we set a minimum score of 80% as a rigorous cutoff for high-quality TFBSs.

### 2.6. Target Gene Prediction of DEMs

The prediction of target genes of DEMs was performed using three computational target prediction algorithms (TargetScan (v.7.0) [31], miRanda (v.3.3) [32], and RNAhybrid (v.2.1.2) [33]). Only when the target gene was identified by all three software was it considered to be the predicted target gene for a given miRNA. The miRNA–gene regulatory network was constructed using Cytoscape (v.3.7.2, http://www.cytoscape.org/, accessed on 18 January 2021) [34] to show the interactions between DEMs and target genes.

### 2.7. Functional Analysis of DEMs

In order to reveal the potential biological functions and principal pathways of DEMs, Gene Ontology (GO) enrichment and Kyoto Encyclopedia of Genes and Genomes (KEGG) pathway analyses were performed for the target genes of DEMs. GO terms were enriched with Database for Annotation, Visualization and Integrated Discovery (DAVID) (https://david.ncifcrf.gov/, accessed on 18 January 2021) [35], which included biological process (BP), cellular component (CC), and molecular function (MF), and KEGG pathway analysis was performed using KEGG Orthology Based Annotation System (KOBAS) (http://kobas.cbi.pku.edu.cn/kobas3/, accessed on 18 January 2021) [36].

### 2.8. Validation of miRNA Expression by Stem-Loop Quantitative RT-PCR

The miRNA-Seq results were validated using RNA samples from C16 and P16 groups with the stem-loop qRT-PCR method [37]. A total of eight miRNAs were randomly selected for qRT-PCR, the primer sequences of which are visible in Table S1 (Supplementary Materials). Reverse Transcription Primers and Quantitative Universal Reverse Primers were provided by the TransScript^®^ miRNA First-Strand complementary DNA (cDNA) Synthesis SuperMix Kit (TransGen Biotech, Beijing, China) according to the manufacturer’s protocols. Next, RT-PCR was performed with SYBR^®^ Premix Ex Taq™ (Toyobo, Shanghai, China) on an ABI PRISM^®^ 7500 Sequence Detection System. Goat U6 snRNA was used as an internal control, and all reactions were done with three technical replicates [38]. The relative expression level of miRNA was quantified relative to the expression level of U6 by using the comparative cycle threshold (2^−ΔΔCt^) method.

### 2.9. FISH Analysis

FISH was performed to detect the location of chi-miR-483 using procedures according to an article published previously [39]. In short, micrometer sections (4 μm thick) were deparaffinized, digested with proteinase K, and hybridized with chi-miR-483 probes labeled with cy3 (red); the negative control was established by replacing the probe with Hybridization Buffer (Servicebio, Wuhan, China). Images were then taken using a positive fluorescence microscope (Nikon, Tokyo, Japan).

### 2.10. Dual-Luciferase Reporter Assay

The fragments containing the putative chi-miR-483 binding sites of wildtype (WT) deltex E3 ubiquitin ligase 3L (DTX3L) 3′UTR and mutant (Mut) were prepared to construct the reporter plasmids and then cloned into the downstream of the luciferase gene in the pGL3-REPORT luciferase vector (Beyotime Biotechnology, Shanghai, China). For luciferase reporter assay, the 293T cells were seeded onto 96-well plates with a density of 10,000 cells/well and transfected with either DTX3L 3′UTR or DTX3L Mut and then with chi-miR-483 mimics and the NC using Lipofectamine 3000 (Invitrogen, Shanghai, China) according to the manufacturer’s protocols. After the transfected cells were harvested at 48 h, the firefly and *Renilla* luciferase activities were measured continuously using a dual-luciferase reporter assay system (Beyotime Biotechnology, Shanghai, China). Lastly, firefly-to-*Renilla* luciferase ratios were calculated for each well, and each measurement was repeated three times in three independent experiments.

### 2.11. IHC Analysis

To determine the expression of DTX3L in the C16 or P16 endometrium, IHC was performed as previously reported [40]. Briefly, sections (4 μm thick) were deparaffinized and blocked with 5% bovine serum albumin (BSA) for 30 min, and they were subsequently incubated with abti-DTX3L rabbit polyclonal antibody (Proteintech, Wuhan, China) at 4 °C overnight. Following incubation with the secondary antibody, the sections were counterstained with hematoxylin and mounted (Fisher Scientific, Shanghai, China). For each sample, a negative control was established using purified nonrelevant immunoglobulin G (IgG). Images were taken with a Nikon 80i microscope (Nikon, Tokyo, Japan). Subsequently, immunohistochemical staining was analyzed by mean integrated optical density (IOD) using ImagePro Plus 6.0 software (Media Cybernetics, Silver Spring, GA, USA).

### 2.12. Statistical Analysis

All experiments were subjected to three independent replicates. Differences in wildtype (WT) or mutant (Mut) DTX3L 3′UTR luciferase reports and the expression level of DTX3L protein under two conditions were compared using Student’s *t*-test (GraphPad Prism version 8.0, San Diego, CA, USA). Values are presented as the mean ± the standard error of the mean (SEM). A value of *p* < 0.05 was considered to be statistically significant.

## 3. Results

### 3.1. Overview of the Sequencing Data

**Figure 1:**
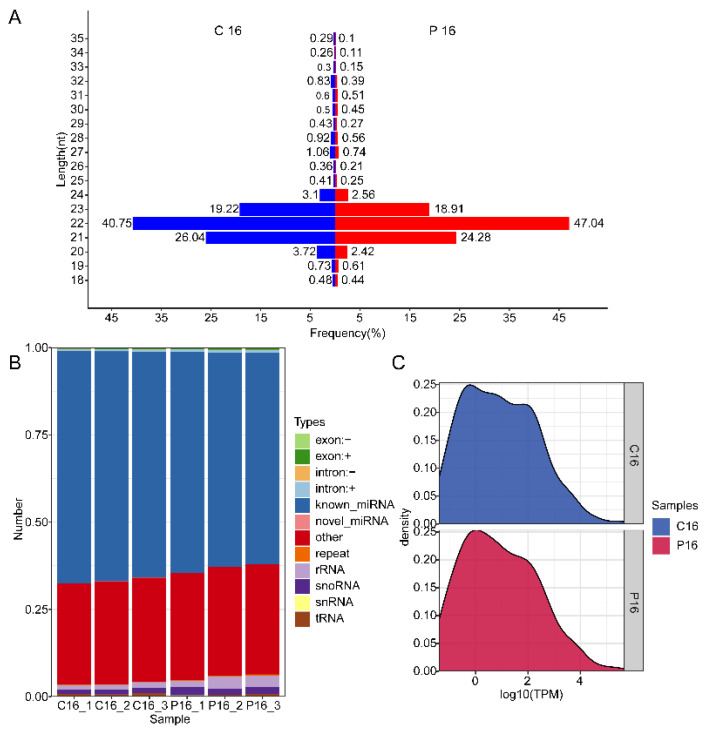
Overview of the sequences generated by miRNA sequencing (miRNA-Seq). (**A**) Sequence length distribution of the sequences generated by miRNA-Seq of the two sets of libraries. The length distribution peaked at 22 nt, i.e., the desired miRNA length. Blue and red represent the results from total sequences obtained from C16 and P16 endometrial samples, respectively. (**B**) Classification of small RNA sequences obtained from individual C16 and P16 endometrial samples. (**C**) The density distribution of miRNA expression. The abscissa represents the value of log_10_ transcripts per million (TPM), and the ordinate represents the corresponding density.

By purifying and sequencing small RNAs from goat endometrium, a comprehensive identification could be accomplished for changes in the expression level of miRNAs in the endometrium of pregnant and nonpregnant goats on day 16 after insemination. After removing low-quality reads and adaptor sequences, 11,966,417 and 13,227,072 raw reads were acquired from the endometrium of C16 and P16, respectively (Table 1). To better assess the changes in sRNAs in the endometrium of C16 and P16, the length distribution of all sRNA reads in the two sets of libraries was surveyed. This length mostly ranged from 21 to 23 nt, and the peak distribution of sequences was 22 nt, which accounted for 40.75% (C16) and 47.04% (P16) of reads (Figure 1A).

From the six libraries prepared from C16 and P16 samples, a total of 11,138,194 and 12,139,519 sRNA sequences were obtained, respectively, for the two conditions. Of these reads, 7,324,385 (65.76%) and 7,500,151 (61.78%) were recognized as known miRNAs in the C16 or P16 library sets, respectively, while 4465 (0.04%) and 3586 (0.03%) were identified as novel miRNAs. The remaining sequences were other RNA types, including rRNA, tRNA, snRNA, snoRNA, exon, intron, and others (Figure 1B; Table S2, Supplementary Materials). The expression level of miRNAs was different in the two conditions (Figure 1C). Despite the Pearson correlation coefficients of different samples within a condition and between conditions being close (Figure S1, Supplementary Materials), the distribution of sRNAs in the two types of samples was different (Figure 1). The high correlation coefficients found between results of the three samples within each condition indicate that these samples were quite homogeneous in terms of their sRNA content.

### 3.2. Analysis of Differentially Expressed miRNAs in C16 and P16 Endometrial Samples

**Figure 2:**
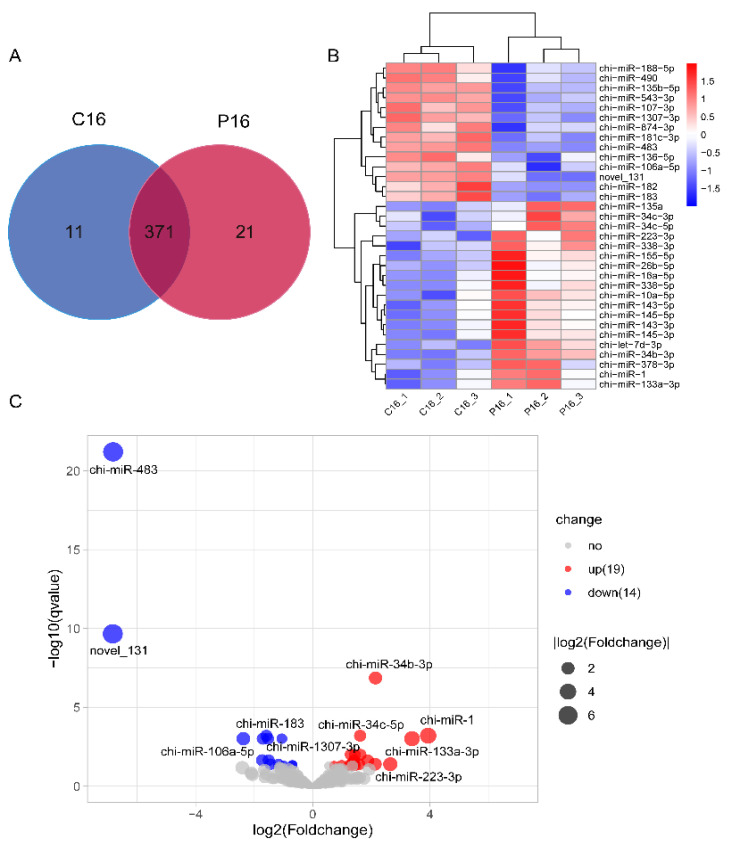
Differentially expressed miRNAs (DEMs) in goat endometrium. (**A**) Venn diagrams of known miRNAs. (**B**) Clustering analysis of DEMs. The color scale is from −2.0 (blue, lower miRNA expression level) to 2.0 (red, higher miRNA expression level). Each row represents one miRNA, and each column represents one sample. (**C**) Volcano plots of DEMs. Each point represents one miRNA. The abscissa represents the value of log_2_ fold-change; the ordinate value represents −log_10_ *q*-value.

To screen the miRNAs in endometrium related to goat endometrial receptivity, the goat endometrium of C16 and P16 was found to present 464 miRNAs, with 403 known miRNAs and 61 novel miRNAs (Table S3, Supplementary Materials). Among the known miRNAs, 371 miRNAs were co-expressed, while 11 and 21 miRNAs were specifically expressed in C16 and P16, respectively (Figure 2A). However, no miRNA was specifically expressed in the novel miRNAs. The 20 most highly expressed miRNAs in C16 and P16 libraries are listed in Table 2.

We focused on miRNAs with *q*-values < 0.05, and 33 differentially expressed miRNAs were chosen, of which, 19 miRNAs were upregulated in the P16 endometrium compared with C16 endometrium in goats, and 14 miRNAs were downregulated (Figure 2C, Table 3). Clustering analysis displayed the expression profile of DEMs (Figure 2B). It is worth noting that the most differentially expressed miRNAs were chi-miR-483 and novel_131, with more than a 100-fold decrease.

### 3.3. Validation of Sequencing Results by qRT-PCR

**Figure 3:**
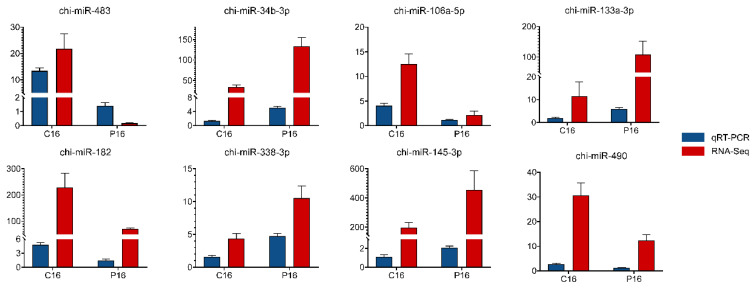
Validation of the expression of miRNAs using qRT-PCR. The relative expression level of miRNA was quantified relative to the expression level of U6 using the comparative cycle threshold (2^−ΔΔCt^) method. Data are displayed as the mean ± standard error of the mean (SEM) values (*n* = 3).

The stem-loop qRT-PCR assay was utilized to mainly detect mature miRNAs. U6 snRNA was chosen as the reference gene. Eight miRNAs were randomly selected for qRT-PCR validation, and the primers used are listed in Table S1 (Supplementary Materials). The validation of the eight selected miRNAs showed that, for all of them, the results of RNA sequencing were very consistent with the results of qRT-PCR (Figure 3).

### 3.4. Potential Transcription Factors Prediction for DEMs

**Figure 4:**
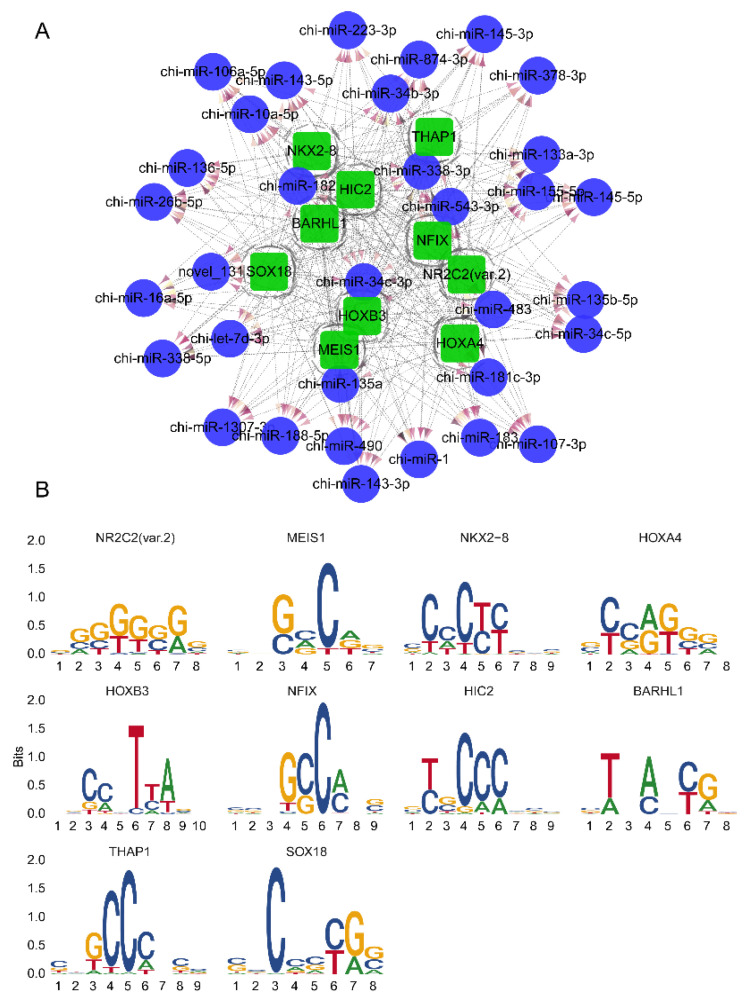
Predicted transcription factors of DEMs. (**A**) The top 10 transcription factors (TFs) with the most TF binding sites (TFBSs). The circles in blue represent DEMs, and the squares in green represent TFs. The dotted line represents the relationship between TFs and miRNAs, and a darker color of the red arrow indicates more TFBSs between the miRNA and TF. (**B**) Binding motifs between the 10 TFs and chi-miR-483.

Herein, potential transcription factors of DEMs were predicted using TFBSTools software. In particular, we identified 253,791 sites where these 33 DEMs could bind to TFs (Table S4, Supplementary Materials). The top 10 TFs with the most TFBSs were *NR2C2* (var.2), *MEIS1*, *NKX2–8*, *HOXA4*, *HOXB3*, *NFIX*, *HIC2*, *BARHL1*, *THAP1*, and *SOX18*, as presented in Figure 4A. In addition, we demonstrate the binding motif between the 10 TFs and chi-miR-483, the most significant differentially expressed miRNA (Figure 4B).

### 3.5. Target Gene Predictions for DEMs

**Figure 5:**
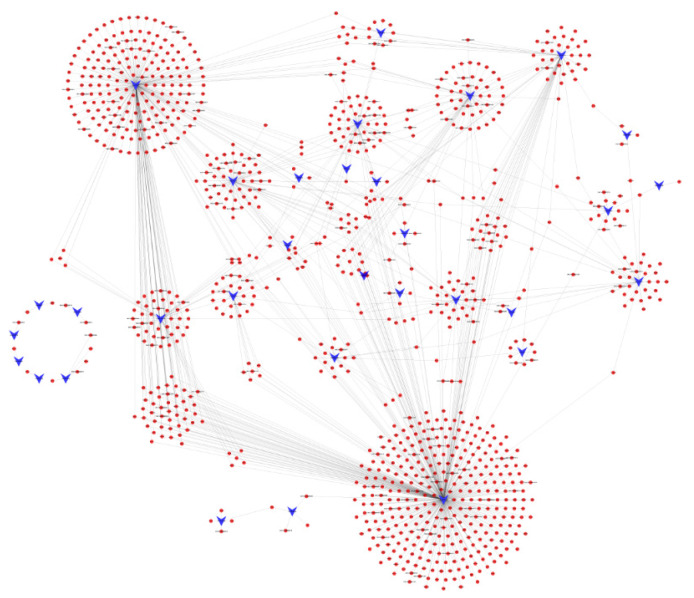
DEM–target gene regulatory network. The circles in red represent target genes and the chevrons in blue represent DEMs.

In animals, miRNAs can downregulate transcript expression levels by interacting with 3′ UTRs, especially complementary sequences of 2–7 nucleotides [41]. When achieving biological process complexity and imperfect complementarity between miRNA and target genes, it is a daunting task to precisely anticipate its target using a single method. As a consequence, three software tools (TargetScan, miRanda, and RNAhybrid) were employed to predict the target genes to confirm accuracy. As a result, 1316 target genes were obtained from the differentially expressed miRNAs, of which 32 were known miRNAs and one was a novel miRNA (Figure 5; Table S5, Supplementary Materials). As displayed in Figure 5, chi-miR-483 and chi-miR-874-3p could regulate abundant genes, and a gene could also be regulated by multiple miRNAs.

### 3.6. Functional Annotation of DEMs in Endometrium

**Figure 6:**
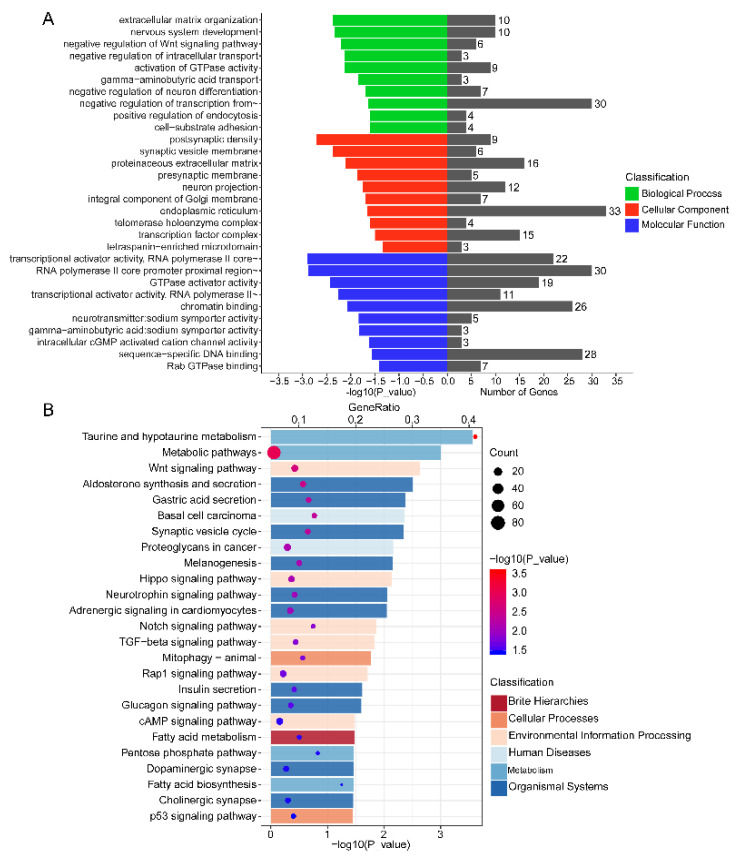
Functional analysis of DEMs. (**A**) Gene Ontology (GO) enrichment analysis of the target genes of DEMs, including biological process, cellular component, and molecular function. (**B**) Kyoto Encyclopedia of Genes and Genomes (KEGG) pathway analysis of the target genes of DEMs.

Understanding the biological functions of DEMs can be accomplished by performing GO enrichment and KEGG pathway analyses on DEM target genes. GO enrichment analysis indicated that target genes were mainly included in biological processes such as extracellular matrix organization, nervous system development, negative regulation of Wnt signaling pathway, negative regulation of intracellular transport, and GTPase activity activation (Figure 6A; Table S6, Supplementary Materials). Moreover, KEGG pathway analysis exhibited that 286 enriched signaling pathways were attained (Table S7, Supplementary Materials). Among the top 25 signaling pathways, the Wnt signaling pathway, Hippo signaling pathway, Notch signaling pathway, Transcription growth factor-beta (TGF-beta) signaling pathway, Rap1 signaling pathway, and p53 signaling pathway significantly influenced endometrium development (Figure 6B).

### 3.7. Chi-miR-483 Can Directly Target the 3′-UTR of DTX3L to Reduce the Expression Level of DTX3L

**Figure 7:**
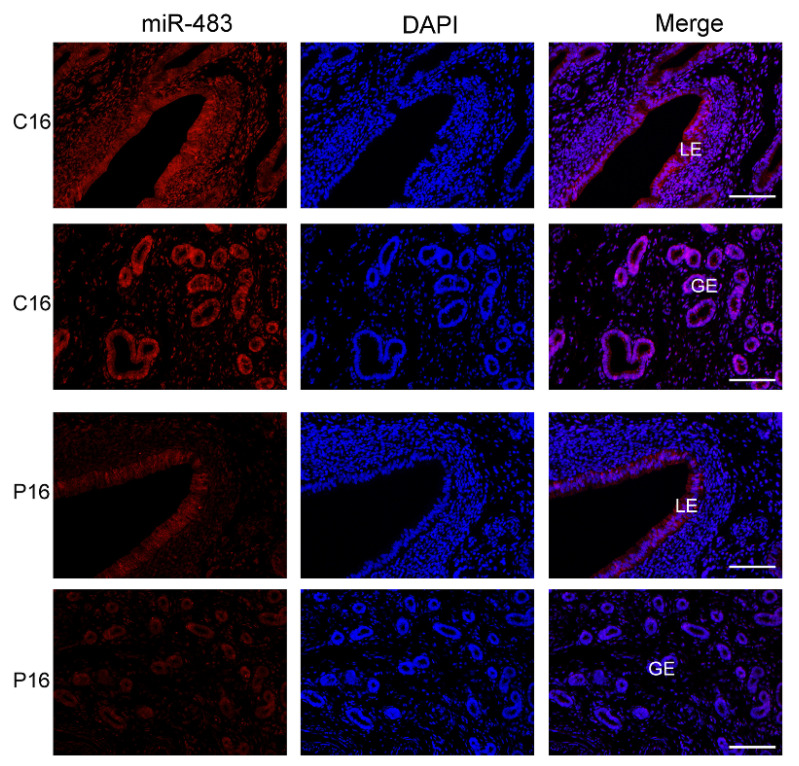
In situ hybridization analysis of chi-miR-483 in the C16 and P16 uterus. The chi-miR-483 was abundantly expressed in the uterine luminal epithelium or glandular epithelium of C16, while it was slightly expressed in P16. The section stained with hybridization buffer without probe was used as the negative control (NC; (Figure S2, Supplementary Materials). Legend: LE, endometrial luminal epithelium; GE, glandular epithelium. Scale bar = 100 μm.

Compared with the nonpregnant goat endometrium on day 16, chi-miR-483 exhibited the most significantly downregulated miRNA. FISH analysis was performed to determine chi-miR-483 location in the C16 or P16 uterus. Interestingly, it was abundantly expressed in uterine luminal epithelium, as well as glandular epithelium, of C16 and slightly expressed in P16, consistent with our above analysis (Figure 7).

**Figure 8:**
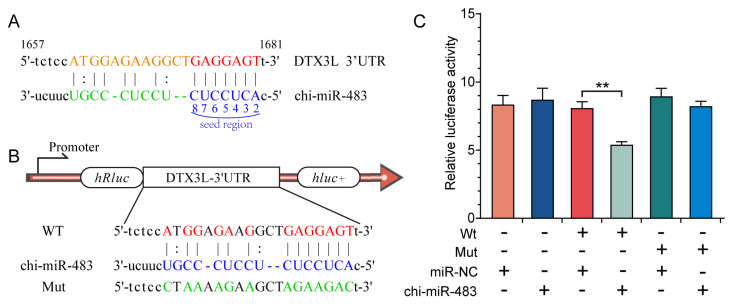
Chi-miR-483 targets the 3′-untranslated region (UTR) of deltex E3 ubiquitin ligase 3L (DTX3L). (**A**) The predicted binding site of chi-miR-483 in the 3′UTR of DTX3L according to bioinformatics analysis. (**B**) Design of the luciferase reporter. WT, the wildtype sequence of DTX3L-3′UTR contains the chi-miR-483 binding site; Mut, the sequence of DTX3L-3′UTR with a mutation in the chi-miR-483 binding site. (**C**) 293T cells were co-transfected with wildtype (WT) or mutant (Mut) luciferase reports of DTX3L 3′UTR with chi-miR-483 mimics or negative control (NC) mimics. The luciferase reporter assay demonstrated that chi-miR-483 significantly decreased the luciferase activity of DTX3L WT in 293T cells. Data are shown as the mean ± SEM values (*n* = 3, ** *p* < 0.01, Student’s *t*-test).

**Figure 9:**
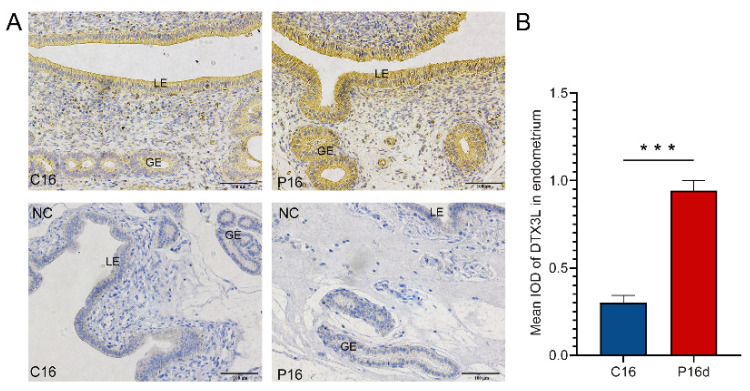
Immunohistochemical analysis of DTX3L in the C16 and P16 uterus. (**A**) Images stained with DTX3L antibodies. The positive signal of DTX3L was distinctly detected in the uterine luminal epithelium or glandular epithelium in P16. The section stained with nonrelevant immunoglobulin G served as the negative control (NC). (**B**) Quantitative analysis of DTX3L by measuring the average integrated optical density (IOD) in the endometrium. Asterisks indicate significant differences (mean ± SEM) between C16 and P16 (*** *p* < 0.001); the *p*-value was determined by Student’s *t*-test. Legend: LE, endometrial luminal epithelium; GE, glandular epithelium. Scale bar = 100 μm.

According to the bioinformatics results of target gene prediction, chi-miR-483 was found to regulate abundant target genes, and this can be revealed by selecting DTX3L as the target gene, exhibiting exact matching with the chi-miR-483 seed sequence (Figure 8A). As presented in Figure 8, compared with the mutant group, the dual-luciferase reporter assay system exhibited a significant reduction in luciferase/*Renilla* luciferase of the wildtype miRNA mimic. However, chi-miR-483 exhibited no remarkable inhibitory impact on the mutant DTX3L 3′UTR dual-luciferase construct (Figure 8B,C). Moreover, the IHC method was deployed to detect the expression levels of DTX3 protein, and they were lower in the uterine luminal epithelium and glandular epithelium of C16 than those of P16, consistent with the specific mechanism of miRNAs (Figure 9). This confirms that chi-miR-483 can directly bind to the DTX3L 3’UTR to reduce the expression level of DTX3L, and it could hold a critical function in forming goat endometrial receptivity.

## 4. Discussion

As a vital factor, endometrium receptivity strongly affects successful embryo implantation and embryonic mortality [3]. The miRNA system regulates numerous biological processes through a single miRNA that can modulate multiple mRNAs after transcription; alternatively, the mRNA can be targeted and regulated by multiple miRNAs [42,43]. Research has proposed that the roles of miRNAs are linked to endometrial receptivity, embryonic development, and implantation [44,45]. Herein, we attempted to expose the miRNA expression profile associated with receptive endometrium formation. However, different cell types in endometrial samples may have heterogeneity. The endometrial receptivity formation caused by differentially expressed miRNAs in different cells may need to be solved by single-cell sequencing technology in the future [46]. Moreover, only a relatively small number of animals were used in this study. Expanding samples for verification in subsequent studies will make the results more reliable.

This study demonstrated that expression levels of chi-miR-1 and chi-miR-133a-3p mostly increased in endometrium on day 16 of pregnancy compared with nonpregnancy, while the expression level of novel-131 and chi-miR-483 mostly decreased. Studies have indicated that reducing the expression level of miR-483 can target the connective tissue growth factor (CTGF) to promote endothelial–mesenchymal transition [47], cell growth, proliferation, differentiation, invasion, and migration, as well as inhibit cell apoptosis [48,49,50,51]. Such a result agrees with the morphological changes of the endometrium during this period in terms of a high level of cell proliferation, migration, and remodeling of tissue structure, thereby attaining endometrium receptivity, leading to successful implantation of the embryo to continue the pregnancy [7]. Moreover, the FISH analysis, dual-luciferase report assay, and IHC analysis confirmed that chi-miR-483 could directly target the 3′-UTR of DTX3L to reduce its expression level, and studies have corroborated that DTX3L can promote cell proliferation, migration, and invasion, as well as play a vital role in establishing pregnancy in cattle [52,53]. This finding advocates that chi-miR-483 holds great promise in forming endometrial receptivity.

Interestingly, previous studies stated that miR-1 and miR-133 are specifically expressed in adult cardiac and skeletal muscle tissues without expression in other tissues [54,55,56,57]. However, we found that chi-miR-1 and chi-miR-133a-3p are overexpressed in the endometrium on day 16 of pregnancy. Studies have confirmed that miR-1 and miR-133 are located on the same chromosomal locus and are transcribed together as a single transcript, resulting in two independent mature miRNAs, which can accomplish diverse biological functions by inhibiting different target genes. For example, miR-1 can promote myogenesis by targeting histone deacetylase 4 (HDAC4), while miR-133 can augment myoblast proliferation by inhibiting serum response factor (SRF) [54]. Moreover, the muscle-specific expression of miR-1/133a exhibits significance in permitting metabolic maturation, as well as proper mitochondrial activity, in skeletal muscles [58]. Furthermore, the two miR-1/133a gene clusters exhibited overlapping functions as inactivation of one or the other cluster would only result in delicate changes in electrophysiological properties of the adult cardiac muscles [59]. Consequently, we speculate that chi-miR-1 and chi-miR-133a-3p, overexpressed in the goat endometrium on day 16 of pregnancy, are most likely to target different genes to realize various physiological roles, with overlapping function between them. Their effects could ultimately promote receptive endometrium formation; however, additional experimental proof is required.

Previous studies revealed that miRNA expression can be regulated by transcription factors [60] predicted to regulate these DEMs. Research has manifested that homeobox-class (HOX-class) homeobox genes are the main candidate genes that regulate endometrial differentiation toward embryo implantation [61]. Moreover, the *HOX* gene exerts its function by acting as a transcription factor and binds to regulatory region of the target gene through its homeobox domain to activate or inhibit transcription [62]. Studies recently demonstrated that some noncoding RNAs (ncRNAs) positioned in the *HOX* locus, including long noncoding RNA (lncRNA) and miRNA, could directly regulate *HOX* gene expression [63]. Therefore, transcription factors may regulate endometrium receptivity by regulating miRNAs, but future robust research is required to confirm this role.

In analyzing differential miRNA target genes, Gene Ontology (GO) analysis affords a convenient and straightforward approach for better understanding the biological functions of genes [64]. Among the top 10 biological processes of GO enrichment, those that drew our interest were extracellular matrix organization (GO: 0030198), negative regulation of Wnt signaling pathway (GO:0030178), activation of GTPase activity (GO:0090630), and cell–substrate adhesion (GO:0031589). As known, embryo implantation comprises extensive tissue remodeling within the endometrium [65], and, since goat exhibits a noninvasive type of implantation, this remodeling is highly significant to produce placental cotyledons and angiogenesis [66]. Through the degradation of the extracellular matrix (ECM), the uterine luminal epithelium experiences a dramatic transformation, producing a receptive endometrium, amenable to receive embryos, proceeding embryo implantation [65,67]. GTPase was recently found to be involved in many biological processes, including the regulation of cell growth, cell positioning, and the cytoskeleton [68,69]. Moreover, studies have showcased that the Wnt signaling pathway is indispensable for developing early embryos and endometrium changes before implantation. Wnt signaling could control endometrial gland formation, and the ablation of specific Wnt signal components would cause implantation failure [70,71], whereas it can also regulate endometrium decidualization [72].

Interestingly, we stated that some biological processes linked to the nervous system were enriched, such as nervous system development (GO:0007399), gamma-aminobutyric acid transport (GO:0015812), and negative regulation of neuron differentiation (GO:0045665). One of the enriched genes, neurotrophin receptor kinase-3 (*NTRK3*), was found to interact with the nerve growth factor (NGF), induce angiogenesis, cell proliferation, and cell adhesion, and regulate gonadal development [73]. This highlights that those genes associated with nervous system development could possess distinguished regulatory functions in other tissues, such as promoting receptive endometrium formation; however, robust supplementary research is required to validate this hypothesis.

Kyoto Encyclopedia of Genes and Genomes (KEGG) pathway analysis is usually employed to get more in-depth insight into interactions between a cluster of genes within biological function scope [74]. The results of this analysis on DEM target genes revealed that some genes might be incorporated in several pathways linked to cancer, including basal cell carcinoma, proteoglycans in cancer, and so on. This results from similarities in cell invasion and angiogenesis between embryo implantation and cancer cell spread [75]. As a consequence, a precise understanding of the endometrium acceptance mechanism could help preclude cancer cell spread. In the top 10 pathways of KEGG analysis, the Hippo signaling pathway is well known as a key regulator of tissue homeostasis, which can govern the size of tissues by regulating cell proliferation, survival, and regeneration [76,77]. Furthermore, the Hippo signaling pathway could regulate the differentiation of endometrial stromal cells in the endometrium [78].

## 5. Conclusions

In summary, Illumina sequencing was utilized to identify 464 unique miRNAs, comprising 403 previously reported and 61 novel miRNAs, from the endometrium of pregnant and nonpregnant goats. In comparison, 33 significantly differentially expressed miRNAs (19 upregulated and 14 downregulated) were identified. The regulatory relationship between miRNAs and upstream transcription factors, the interaction analysis of miRNAs and their target genes, GO enrichment, and KEGG pathway analysis could provide a better understanding of how miRNAs mediate target gene regulation in endometrium receptivity formation. Taken together, these findings present new insights into the role of miRNAs in regulating goat endometrium receptivity.

## Figures and Tables

**Table 1:** Summary of high-throughput sequencing in C16 (day 16 of the estrous cycle) and P16 (day 16 of pregnancy) endometrium of goats.

Sample	Total Reads	N% > 10%	Low Quality	5′ Adapter Contaminant	3′ Adapter Null or Insert Null	With Poly A/T/G/C	Clean Reads
C16	12,151,788	18	11,569	679	151,535	21,570	11,966,417
	(100.00%)	(0.00%)	(0.09%)	(0.01%)	(1.25%)	(0.18%)	(98.47%)
P16	13,520,599	52	15,103	1628	265,879	10,864	13,227,072
	(100%)	(0.00%)	(0.11%)	(0.01%)	(1.97%)	(0.08%)	(97.83%)

N: A base whose base information cannot be determined; low quality: the number of bases with Phred value ≤ 20 in a single-ended read exceeds 30% of the total number of bases in the read; with poly A/T/G/C: continuous A/T/G/C bases in reads.

**Table 2:** Top 20 miRNAs in C16 and P16 endometrium.

miRNA	Average TPM in C16	miRNA	Average TPM in P16
chi-miR-148a-3p	470,173	chi-miR-148a-3p	358,978
chi-miR-99a-5p	243,974	chi-miR-99a-5p	239,837
chi-miR-143-3p	46,655	chi-miR-143-3p	110,970
chi-miR-21-5p	38,853	chi-miR-26a-5p	53,046
chi-miR-26a-5p	33,294	chi-miR-21-5p	37,842
chi-miR-126-3p	19,458	chi-miR-10b-5p	20,501
chi-miR-10b-5p	15,653	chi-miR-126-3p	17,344
chi-let-7i-5p	9218	chi-let-7f-5p	12,137
chi-let-7g-5p	7995	chi-let-7g-5p	11,049
chi-let-7f-5p	7944	chi-let-7i-5p	10,918
chi-miR-199a-3p	6749	chi-let-7a-5p	7798
chi-let-7a-5p	5646	chi-miR-27b-3p	7696
chi-miR-200a	5589	chi-miR-1	7249
chi-let-7c-5p	5033	chi-miR-199a-3p	6988
chi-let-7b-5p	4965	chi-let-7c-5p	6668
chi-miR-27b-3p	4601	chi-let-7b-5p	6219
chi-miR-151-3p	4501	chi-miR-10a-5p	5917
chi-miR-200b	4439	chi-miR-151-3p	5227
chi-miR-125b-5p	3800	chi-miR-200a	5189
chi-miR-10a-5p	3327	chi-miR-200b	4371

**Table 3:** The differentially expressed miRNAs C16 and P16 endometrial samples.

miRNA	Average Readcount in C16	Average Readcount in P16	log_2_ Fold-Change	*p*-Value	*q*-Value
novel_131	39.85	0.35	−6.83	1.29 × 10^−12^	2.18 × 10^−10^
chi-miR-483	152.43	1.35	−6.82	1.83 × 10^−24^	6.16 × 10^−22^
chi-miR-106a-5p	87.16	16.88	−2.37	2.71 × 10^−5^	9.50 × 10^−4^
chi-miR-188-5p	39.37	11.93	−1.72	1.12 × 10^−3^	2.21 × 10^−2^
chi-miR-135b-5p	51.37	15.79	−1.70	2.16 × 10^−5^	9.50 × 10^−4^
chi-miR-183	932.05	311.26	−1.58	9.34 × 10^−6^	6.13 × 10^−4^
chi-miR-182	1595.76	553.28	−1.53	3.13 × 10^−5^	9.58 × 10^−4^
chi-miR-181c-3p	32.80	11.56	−1.50	1.03 × 10^−3^	2.17 × 10^−2^
chi-miR-874-3p	40.21	14.78	−1.44	2.94 × 10^−3^	3.96 × 10^−2^
chi-miR-490	212.90	94.84	−1.17	2.57 × 10^−3^	3.94 × 10^−2^
chi-miR-1307-3p	454.00	218.84	−1.05	2.82 × 10^−5^	9.50 × 10^−4^
chi-miR-136-5p	1387.52	693.62	−1.00	4.80 × 10^−3^	4.90 × 10^−2^
chi-miR-543-3p	441.79	272.33	−0.70	3.46 × 10^−3^	4.32 × 10^−2^
chi-miR-107-3p	457.79	284.97	−0.68	3.46 × 10^−3^	4.32 × 10^−2^
chi-miR-135a	269.47	446.58	0.73	4.78 × 10^−3^	4.90 × 10^−2^
chi-miR-10a-5p	23116.13	45875.93	0.99	3.90 × 10^−3^	4.53 × 10^−2^
chi-miR-26b-5p	9990.30	23746.46	1.25	3.82 × 10^−3^	4.53 × 10^−2^
chi-let-7d-3p	31.10	75.94	1.29	3.38 × 10^−4^	9.49 × 10^−3^
chi-miR-16a-5p	1380.89	3376.81	1.29	4.57 × 10^−3^	4.90 × 10^−2^
chi-miR-145-3p	1364.07	3536.32	1.37	1.51× 10^−3^	2.68 × 10^−2^
chi-miR-143-3p	325393.81	862304.74	1.41	2.13 × 10^−3^	3.59 × 10^−2^
chi-miR-338-3p	30.42	81.38	1.42	9.74 × 10^−4^	2.17 × 10^−2^
chi-miR-378-3p	1175.13	3190.20	1.44	4.14 × 10^−4^	9.97 × 10^−3^
chi-miR-143-5p	1553.50	4241.32	1.45	4.04 × 10^−3^	4.54 × 10^−2^
chi-miR-145-5p	7862.77	23587.26	1.58	2.81 × 10^−3^	3.96 × 10^−2^
chi-miR-34c-5p	1040.08	3191.46	1.62	9.38 × 10^−6^	6.13 × 10^−4^
chi-miR-155-5p	297.21	916.87	1.63	4.04 × 10^−4^	9.97 × 10^−3^
chi-miR-34c-3p	19.36	71.16	1.88	1.27 × 10^−3^	2.38 × 10^−2^
chi-miR-338-5p	12.77	55.77	2.13	2.84 × 10^−3^	3.96 × 10^−2^
chi-miR-34b-3p	233.76	1032.78	2.14	1.23 × 10^−9^	1.38 × 10^−7^
chi-miR-223-3p	1.88	11.77	2.65	2.54 × 10^−3^	3.94 × 10^−2^
chi-miR-133a-3p	80.34	842.29	3.39	2.71 × 10^−5^	9.50 × 10^−4^
chi-miR-1	3678.63	56458.03	3.94	1.09 × 10^−5^	6.13 × 10^−4^

## Data Availability

The datasets presented in this study can be found in online repositories. The raw reads produced in this study were deposited in the NCBI Sequence Read Archive (SRA), accession number PRJNA693422.

## Supplementary Materials

The following are available online at https://www.mdpi.com/2218-273X/11/3/472/s1, Figure S1. Pearson correlation between samples; Figure S2. Negative control for fluorescence in situ hybridization; Table S1/ Primers used for the miRNA validation by RT-qPCR; Table S2. Classification of small RNA sequences obtained from individual C16 and P16 endometrial samples; Table S3. TPM values of identified miRNAs in the endometrium of pregnant and nonpregnant goats; Table S4. Predicted transcription factors of DEMs; Table S5/ Target genes of differentially expressed miRNAs; Table S6. GO enrichment analysis of the target genes of DEMs; Table S7. KEGG pathway analysis of the target genes of DEMs.
