# Supplementary figures and images for: Differential MicroRNA Expression Involved in Endometrial Receptivity of Goats
Source: Biomolecules. 2021 Mar 22;11(3):472. doi: 10.3390/biom11030472 (PMC8004627; doi:10.3390/biom11030472)

Pearson correlation between samples

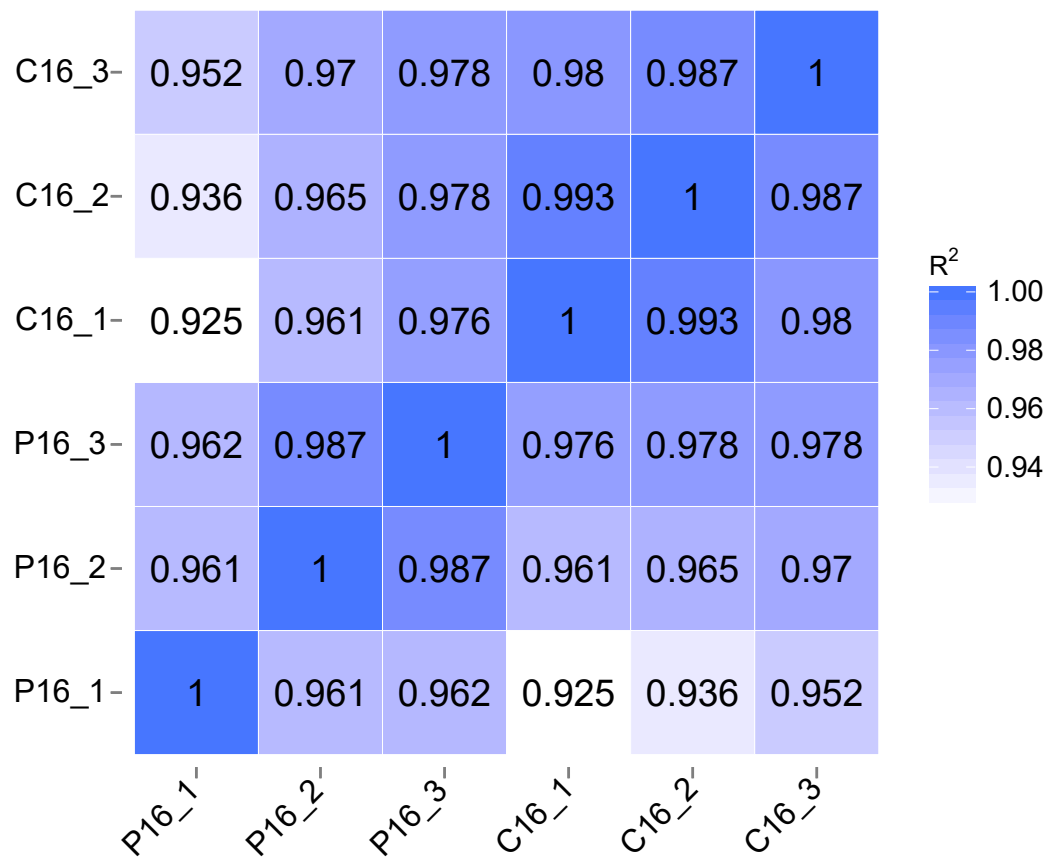

Supplement: Supplementary file 1 [file biomolecules-11-00472-s001.zip › Supplementary Figure S1.pdf]

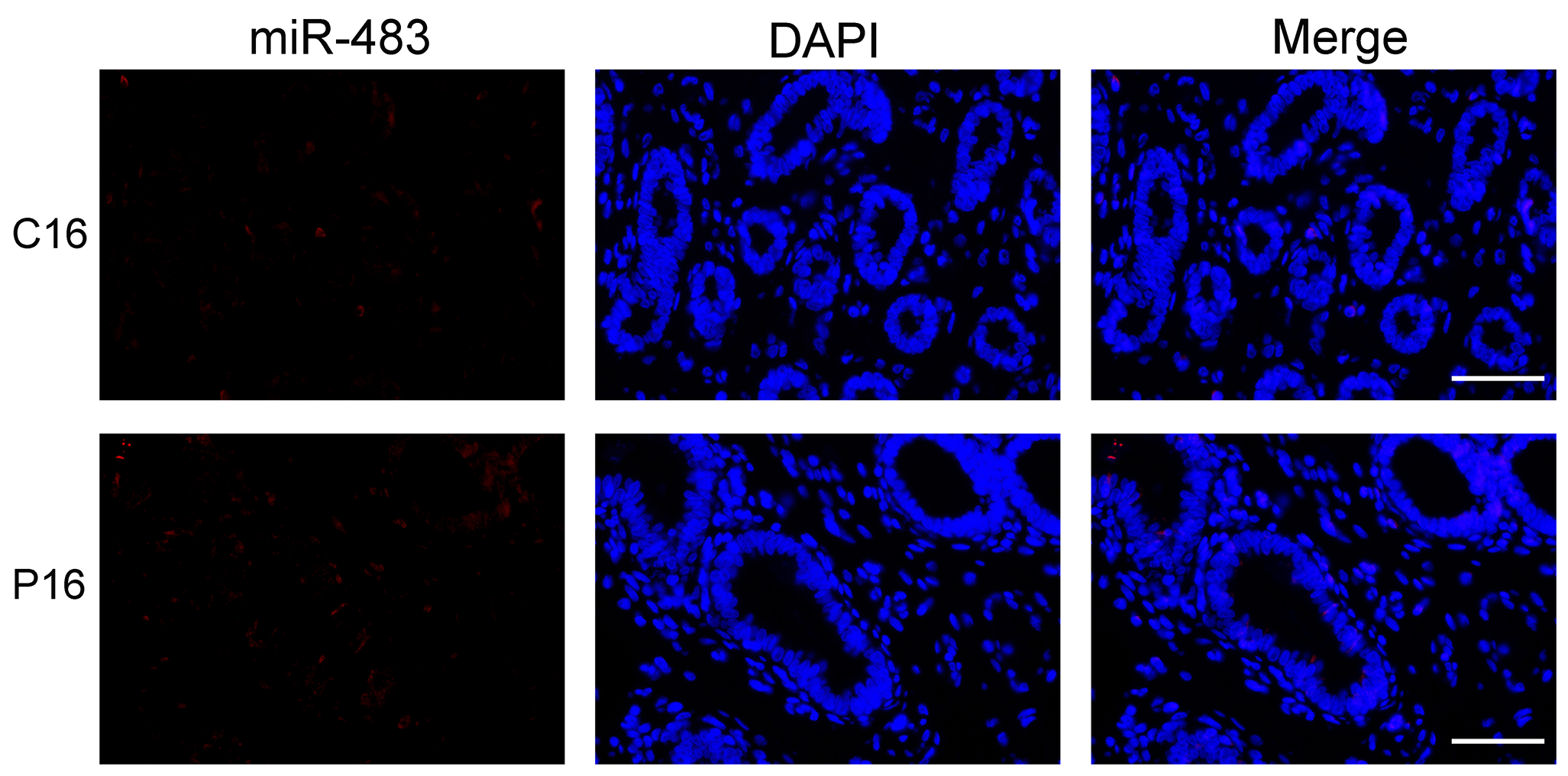

Supplement: Supplementary file 1 [file biomolecules-11-00472-s001.zip › Supplementary Figure S2.tif]
